# How Do Government Policies Promote Green Housing Diffusion in China? A Complex Network Game Context

**DOI:** 10.3390/ijerph19042238

**Published:** 2022-02-16

**Authors:** Xia Cao, Tianjiao Zhao, Zeyu Xing

**Affiliations:** 1 School of Economics and Management, Harbin Engineering University, Harbin 150001, China; caoxia@hrbeu.edu.cn; 2 School of Management, Zhejiang University of Technology, Hangzhou 310023, China; xingzeyusmile@zjut.edu.cn

**Keywords:** greening housing, government policies, GH diffusion, complex network

## Abstract

To reduce energy consumption and environmental pollution in the construction industry, many countries have focused on the development of green housing (GH), which is a type of green building for residential use. In China, the local governments have introduced various incentive policies to encourage the development of GH; however, its scale is still small and unevenly distributed. This implies a necessity to optimize the policies that apply to the GH incentive. To promote GH diffusion, we built an evolutionary game model on a complex network to analyze the impacts of government policies on GH pricing and demand and the profits of real estate enterprises developing GH. By implementing simulations, we further explored the incentive effect and operational mechanism of the government policies. The results show that the subsidy policy, the preferential policy for GH, and the restriction policy for ordinary housing can effectively promote the diffusion of GH to 0.6752, 0.506, and 0.5137 respectively. Meanwhile, the incentive effect of the enterprise subsidy policy and GH preferential policy gradually decreases with the increase in policy strength. In terms of the demand side, the consumer subsidy policy could promote GH diffusion to 0.7097. If the subsidy is below 120 CNY/m^2^, the effect of the consumer subsidy policy is less powerful than that of the enterprises subsidy policy; conversely, the former is slightly more effective than the latter. The outcome of the study has managerial implications on governmental decision-making, especially on the strategy design of incentive policies for GH.

## 1. Introduction

Green housing is a special kind of green building for residential use, the characteristics of which are maximum savings on resources, protection of the environment and reduced pollution [1,2]. GH can provide a healthy and comfortable living space while minimizing energy consumption and carbon emissions during construction and living, for innovation and improvements in design planning, material selection and construction technology [3]. Compared with ordinary housing (OH), GH has obvious advantages in reducing carbon emissions, lowering energy consumption, protecting the ecological environment and improving people’s living conditions [4,5]. In order to better regulate the development of green buildings, countries around the world have developed relevant certification standards, such as LEED in the US, BREEAM in the UK, DGNB in Germany, CASBEE in Japan, GB Tool in Canada, Green Star in Australia, HQE in France and ESGB in China. For now, as an effective way to reduce energy consumption and environmental pollution in the construction industry, GH is becoming a major trend in the decarbonization of the real estate market. Therefore, many countries around the world have attached great importance to the development of GH. 

In China, to solve the problem of high energy consumption in the construction industry, the Chinese government has introduced and promoted the concepts of sustainable buildings, eco-buildings etc., since the end of the last century, and made green buildings the main development direction around 2000. In 2006, China launched the first ‘Green Building Evaluation Standard’; since then, documents such as the ‘Green Building Action Plan’ have been formulated to plan, guide and promote the development of the green building industry, leading to a significant increase in the overall scale of green buildings, whereas the development of GH was slow. The overall scale of GH is small and accounts for a relatively low proportion of green building stock overall; based on the latest available data, as of September 2016, there were a total of 4515 green building projects in China, of which only 56 were residential projects, all accounting for only around 1%. In terms of floor area, as of 2017, less than 0.4% of China’s overall building stock was green, and in 2018, less than 1% of new residential space was green [6]. Meanwhile, the development of GH across China’s provinces has varied widely, showing an uneven geographical distribution; the number of GH projects and their scale in terms of construction area in the coastal provinces in the southeast far exceed that in the provinces in the northeast and northwest regions [7]. In addition, the number of GH structures with the high-grade green building label is also small; in 2018, a total of 113 green building projects in China were awarded the three-star green building label, exceeding 100 for the first time, but only seven were residential projects. Compared to developed countries, further incentives are needed for GH in China; for the local governments, especially in the low-performing provinces, that means a requirement for more efficient and appropriate policies [8]. Hence, we need to clarify the influence mechanism of existing policies to optimize their design and implementation and accomplish the development goals of GH.

Existing research suggests that government policy is an effective external incentive that can weaken barriers to GH development, but most studies merely view the policy as an influencing factor [9] with an impact on the decision-making of real estate enterprises [10,11] and consumer purchase intentions [12,13]. 

Few of them have studied the scope of the impact and the operational mechanism of the policies. To address these issues and the shortcomings of existing research, this paper focuses on an analysis of the effect of policy on the diffusion of GH among real estate enterprises in China. Thus, we summarized and classify the existing incentive policies for GH and determine market demand, optimal pricing and enterprises’ profits from GH and OH based on consumer utility. Then, we constructed an evolutionary game model for real estate enterprises on a scale-free network, in which the decision to develop GH is mainly influenced by market supply and demand and government policies. The enterprises learn other’s strategies by comparing their returns under the condition of limited rationality, and the strategy of developing GH within the enterprise group is continuously spread in the game process. The general idea of the research in this paper is as follows: Section 2 reviews relevant literature. Section 3 builds the network evolution game model of GH diffusion. Section 4 simulates the impact of government policies on GH diffusion. Finally, the research conclusions and policy recommendations are given in Section 5.

**Figure 1:**
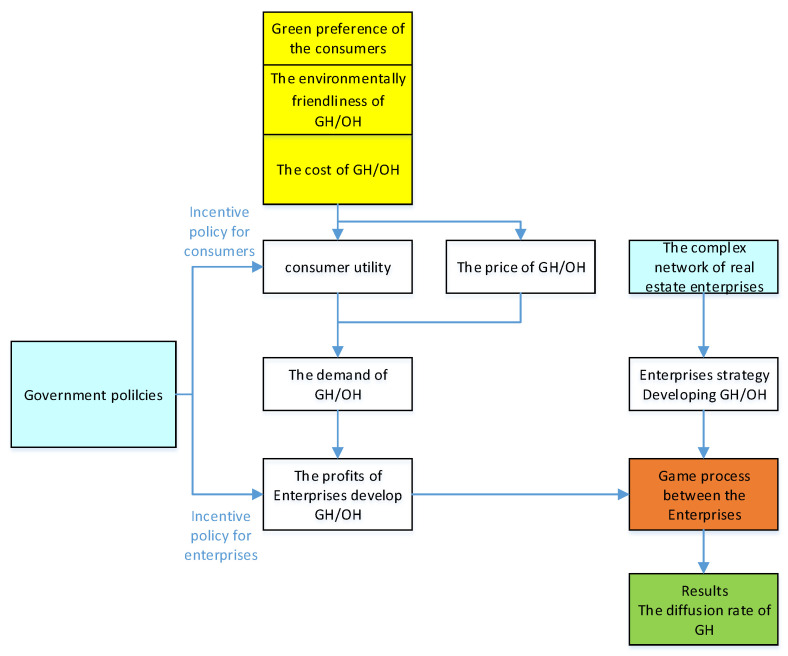
Analytic framework.

Based on the above background, the analytic framework of the paper is as shown in Figure 1. The main objectives of this paper are to determine the effects of government policies on enterprises’ decisions to develop GH, and how to use those effects to promote the diffusion of GH. In this way, this study seeks to reveal the incentive level and operational mechanism of the different policies, and then, to provide some suggestions for the growth of GH.

The main novelty of this paper is as follows. (1) An evolutionary game model on a complex network constructed of real estate enterprises is developed, providing a new approach to examine the impact of government policy on business decisions. (2) The diffusion process of GH under the different incentive policies is simulated on the complex network. This is helpful to analyze the impact of the different types and intensity of policies on green housing market prices, demand, and corporate profits, thus providing a new perspective to explain the incentive effect and operational mechanism of government policies on GH diffusion.

The findings of the paper will reveal which policies are more effective in increasing the willingness of companies to develop green housing, and how to implement the various incentives with the right intensity, which will provide valuable recommendations for policy makers, as well as a reference for other developing countries on how to use policies to encourage green housing development.

## 2. Literature Review

### 2.1. The Impact of Government Policies on GH Diffusion 

As a special type of green building, most GH has a significantly commercial attribute, which means the development of GH should rely on real estate enterprises. The enterprises will only take the initiative to invest in GH if the investment returns are equal to or higher than those of OH [2]. However, in order to meet the evaluation standards of green buildings, the design, material selection and construction techniques involved in green housing need to meet higher requirements, which makes GH more expensive to build [14], while the green transaction of ordinary housing also requires additional retrofitting costs [15] that also make green housing more risky in the market [6]. These factors have to some extent hindered the development of green housing, and difficulties in access to capital and information have also reduced the willingness of real estate companies to develop GH [16]. Meanwhile, customers, due to a lack of green concepts and knowledge, have little preference for GH [17]. Thus, in the absence of government incentive policies, the supports and demands for GH are weak, and only if the costs and benefits are changed can the willingness to develop or purchase GH be enhanced [18]. Thus, government policies can significantly influence the behavioral decisions of various stakeholders of GH [19], especially developers and buyers, by using direct financial subsidies, tax breaks, credit supports, etc. [20,21], which also affect the price premium from environmental certification label and market diffusion of GH [22,23].

A positive attitude of the government is necessary for the implementation of GH incentives, and along with the provision of subsidies, regulation of the GH market is also necessary [24]. To this end, a comprehensive policy system is needed to promote the development of GH, and this system should include all aspects of planning, implementation, monitoring, and evaluation of GH [25]. Additionally, when formulating specific policy measures, the government should draw on past historical experience in a limited way to avoid forming path dependency. At the same time, it should adapt to local conditions, enhance policy flexibility, make trade-offs between policy costs and environmental benefits, and avoid over-reliance on government incentives on the supply side [26,27]. Instead of relying solely on regulation, market expectations should be adjusted to levels that improve the technical capacity of the industry, guide consumers, and incentivize early adopters to enhance the initiative of market players in choosing GH [28,29], so as to establish a holistic and scientific incentive policy system. 

At present, many countries have implemented GH incentives [16]: for example, tax credits in the United States and Spain, the Green Mark incentive program in Singapore, and subsidies in New Zealand. With different incentive models, the effectiveness of the implementation of these policies in different political, environmental and social contexts also has varied considerably [30]. The effects of government policies are influenced by factors such as incentive patterns, market demand, and developer preferences, which may vary by region. For real estate enterprises, some scholars have analyzed the key factors affecting the development of GH by real estate enterprises and concluded that government incentive policies should be planned and formulated around factors such as ‘the level of local economic development, development strategies and innovation orientation, developers’ knowledge and positioning of GH, and experience and capability of GH’ [31].

In terms of specific incentive models, non-financial subsidy policies for real estate enterprises, including floor area ratio incentives, can also achieve better results [32]. Meanwhile, reputational and fiscal incentive policies are effective ways to promote behavioral intentions and actual behavior of the enterprises adopting GH [6,19]. For the consumer, some scholars argue that demand-pull policies are more effective in the promotion of green homes [33]. Therefore, the government should pay attention to the needs and preferences of GH customers when implementing green building incentives [34]. In addition, some scholars have studied GH incentive models, financial subsidy approaches, and technological upgrades from the perspectives of transaction costs, green finance [35], and regional economic fundamentals [36] and population composition [37]. Some scholars have also studied the influencing factors of GH incentive policies. They found that the incentive effect of subsidy policy increases significantly with the increase in consumer green preferences and the potential benefits to developers [38,39]. 

In general, the existing literature on GH policies focuses on the necessity of policy implementation, types and models of policies, and the effects or impacts from implementation of incentive policies. Considering that the competitive relationship among enterprises reflected a network structure, this paper builds a network evolutionary game model to study the impact of GH incentive policies on the decision-making behavior of real estate enterprises and provides a new perspective to explain the incentive effect and operational mechanism of government policies on GH diffusion. 

### 2.2. The Application of Evolutionary Game of Complex Networks

Evolutionary game theory is developed on the basis of traditional game theory by introducing biological evolution theory. The theory is concerned with the interaction between different players or groups, and assumes that the player of the game has ‘finite rationality’ and can learn the optimal game strategy through ‘replication dynamic mechanism’. The mechanism of interaction between the players and the influence of various factors can be studied by analyzing the change in the proportion of a strategy adopted by a group in the game process [40]. This model has certain advantages in studying the behavioral decisions of organizations, firms, or individuals, which has led to its wide use in many fields such as economics, management, and organizational behavior. Some scholars have studied the promotion of green buildings with the help of evolutionary game models; Liu (2016) analyzed the influence of incentive policies in different stages of green building development by forming a dynamic evolutionary game model and concluded that the incentive and constraint policies on green buildings in different developmental stages should be focused and adjusted according to the market environment [41]. Qian (2015) studied how to increase developers’ willingness to build GH through policy incentives by analyzing the evolution of the game strategies of the government and developers [15]. Fan (2018) further studied the decision-making mechanisms of the government and developers in the dynamic game process and found that under the interaction between the government and developers, the price premium, the level and affordability of incentives are the key factors influencing the player’s decisions [42]. Feng (2020) developed an evolutionary game model between developers and consumers using government subsidies as a control factor and proposed that providing government subsidies to construction units can promote the development of green buildings [43].

By using evolutionary game models, scholars’ research has revealed to a certain extent the evolutionary regularity of game relationships and behavioral decisions among major stakeholders, such as government, developers and consumers in the process of green building development. However, the general evolutionary game model does not consider the interrelationships among individuals within the group, while in reality, many social systems have the topological structure and statistical characteristics of networks, and the evolutionary game of individuals within the system is closely related to the network structure [44]. Scholars have further studied the evolutionary game on complex networks, in which individuals adjust their own game strategies by comparing their gains with neighboring nodes, thus reflecting a change in game strategy within a group at the macro level. The process of adjusting business strategies in a real market environment is complex and subject to many factors, such as the size of the company, its business philosophy and profitability. However, with the help of complex network evolutionary game models and simulation techniques, we can analyze the influence of various factors on the evolution in strategies of the game players and how this influence spreads across the complex network.

At present, only a few scholars have used complex network evolutionary game models to explore the diffusion of low-carbon or green technology innovation [45,46]. Wu (2017) established a low-carbon evolutionary model based on the game between government and firms in a complex network and found that firms’ expectations of government subsidies, beyond vision and other incentives, determine whether low-carbon strategies can feasibly spread and the speed of their diffusion [47]. Wang (2019) investigated the diffusion of low-carbon technologies from the perspective of network characteristics and consumer environmental sensitivity by modeling the network evolution game among firms. The results show that increasing the linkages between firms helps to promote the diffusion of low-carbon strategies, while the diffusion of low-carbon technologies will increase significantly with the increase in consumers’ environmental sensitivity [48]. Zhang (2019) constructed a technology diffusion model based on alliance firms using complex networks and evolutionary games to investigate the effectiveness of low-carbon policies in promoting the diffusion of green technologies in alliance firms. The results show that carbon trading markets, environmental protection taxes and innovation subsidies have significant positive effects on accelerating technology diffusion among firms, but that the implementation of different levels of policies (especially market-based policies) leads to different diffusion rates [49]. In general, when low-carbon or green technologies are diffused in a complex social system, it is not only necessary to consider the spatial structure formed by the interaction between participants, but also to pay attention to the influence of the network environment on the strategy choice of game individuals [37]. 

The above literature summarizes the application experience of evolutionary game models on complex networks, analyzes the influence of government policies on enterprises’ decisions, reveals the incentive mechanism of the low-carbon or green technology incentive policies, and provides a theoretical basis for the improvement of government industrial policies. However, when discussing the effect of government policies, most of the existing literature does not discuss the changes in green product prices, market demand and corporate profits, nor does it fully consider the effect of competition among enterprises on the effect of policy incentives. As for the policy of GH, the existing studies mainly summarize and analyze the existing incentive policies of GH in China, and discuss the incentive model, financial subsidies, technology upgrading, and information disclosure from the policy level. There is a lack of research on the mechanism and utility analysis of government incentive policies, and the research methods are mainly based on questionnaires and data analysis. Therefore, this paper uses the evolutionary game model on complex networks to analyze the effects of various incentive policies on GH prices, market demand, and enterprises’ profits, discusses the incentive mechanisms and implementation effects of different types of incentive policies on the diffusion of GH through simulations, and then proposes countermeasures.

## 3. Proposed Model and Method

### 3.1. Problem Description and Hypothesis

In 2019, China revised its green building evaluation standards, which classify green buildings as basic, one-star, two-star and three-star, and increased requirements for the habitable attributes of green buildings. In 2020, China’s latest 14th Five-Year Plan proposal demanded that local governments ‘accelerating the promotion of green and low-carbon development, strengthen the spatial planning and use control of land, and make efforts to develop green building’. On the basis of the central government’s policies, local governments at the provincial level made the policies more refined and specific, including land transfer, land planning, financial subsidies, tax reduction, loan support, floor area ratio incentives, urban infrastructure supporting fees reduction, the priority of administrative approval and awards, the priority of enterprise qualification approval and promotion, and consumer guidance. Most of these policies are applicable to the GH market and can be divided into incentive policies for real estate enterprises and incentive policies for consumers according to the target audience [37,39], and into financial and non-financial subsidy policies according to the mode of action [30,50,51]. The classification of green building incentives by these scholars is highly representative and offers a more flexible approach to their classification. Based on relevant studies by previous scholars and considering the policy targets and modes, we classify the incentive policies for GH into the following four categories.

First, subsidy policy for the enterprises

The subsidy policies for real estate enterprises developing GH can be divided into direct and indirect subsidy policies based on whether fiscal funds are involved. Among them, direct subsidy policies with clear implementation standards mainly include monetary subsidies, tax reduction, and urban infrastructure facility fee reduction. In Beijing, Tianjin, Shanghai, Jiangsu, Shandong, Guangdong, Chongqing, Shaanxi and other provinces, the monetary subsidy is mainly to directly issue subsidies to enterprises according to green building grades obtained, construction area or project type. Shanghai, Fujian and other regions have further formulated tax reduction policies for green residential projects. Provinces such as Inner Mongolia, Hainan, and Qinghai have reduced the urban infrastructure facility fee for GH projects in varying ranges. Indirect subsidy policies are mainly based on floor area ratio incentives and preferential loan interest rates. Zhejiang, Fujian, and Guizhou use floor area ratio incentives to reduce the cost to the real estate enterprises developing GH. In addition, Beijing, Henan, Anhui and other provinces require banks to give priority to GH development companies for preferential interest rates. Among them, Anhui has clearly stated that the loan interest rate for enterprises developing GH can be lowered by 1%.

Second, preferential policies for GH

The preferential policies for GH mainly include the priority of administrative approval and awards for GH projects, and the priority of enterprise qualification approval and promotion for enterprises developing GH. Some provinces, such as Fujian, Inner Mongolia, Hubei, Hunan, Qinghai, Ningxia, etc., have established a rapid approval mechanism for green buildings to accelerate the construction and sales of green buildings. In terms of project awards, Shandong, Inner Mongolia, Henan, Shaanxi, Anhui, Jiangxi, Hubei, Hunan, Guangxi, Chongqing, Guizhou, Ningxia and other provinces have made green building projects a priority or necessity for the selection of various national or provincial building awards, which will strengthen the influence of green buildings in the industry by focusing on the advantages of industry evaluation. In terms of enterprise qualification approval, provinces and cities such as Beijing, Hubei, Hunan, Inner Mongolia and Jilin have proposed to reward enterprises that have achieved significant results in implementing green buildings with exemptions, priority and extra points in the annual inspection of enterprise qualifications, upgrading of qualifications and renewal of certificates, and bidding for projects. Compared to the enterprise subsidy policy mention above, the preferential policy cannot bring direct financial benefits to the enterprises, but it can increase the reputation of the real estate enterprises and reduce the administrative costs or land costs associated with GH. The implementation of preferential policy will enhance the competitive advantage of GH projects and will be transformed into a long-term benefit for real estate enterprises [52,53], but the competitive advantage only exists when the rival is OH; if the competitor enterprise chooses to develop GH, the benefits due to the policy will be weakened or invalidated.

Third, restriction policy for OH

Currently, China does not have a punitive policy for the development of ordinary residential buildings, but the existing incentive policy for green residential buildings objectively has a limiting effect on the development of ordinary residential buildings. In July 2020, the Ministry of Housing and Construction and the Development and Reform Commission and other departments jointly issued the Action Plan for the Creation of Green Buildings, which requires that by 2022, the proportion of green floor space in new buildings in cities and towns will reach 70% in that year. Previously, many provinces and cities also had clear requirements for the proportion of GH in new urban construction. The implementation of relevant land planning policies and the setting of targets for the scale of green building development, to a certain extent, restricted the supply of land for OH, leading to an increase in the cost of developing OH for enterprises [16]. At the same time, there are also specific requirements for energy efficiency in ordinary residential buildings in some provinces; for example, the Implementation Plan for Green Building Action in Guizhou Province requires that new buildings in urban areas strictly implement mandatory energy efficiency standards and to meet 100% of the standards at the design stage. In addition, since 1 January 2018, China has officially implemented the Environmental Protection Tax Law, which imposes taxes on four major pollutants, including air, water, solid waste and noise; the introduction and implementation of the environmental protection tax has also increased the cost of developing OH for enterprises to a certain extent. Overall, these policies have increased the cost of developing OH for real estate enterprises, negatively impacting their willingness to develop OH.

Fourth, subsidy policy for the consumers 

Currently, only a few provinces in China have implemented incentives for consumers to purchase GH; policies are relatively limited and focus on providing direct or indirect subsidies for buyers, including monetary subsidies, lower interest rates or higher loan amounts, and tax rebates [37]. Jiangsu and Shaanxi have provided monetary subsidies to encourage consumers to purchase GH. Zhejiang, Shaanxi and Jiangsu have offered larger loan amounts to consumers who use their provident funds to purchase GH, and Anhui has required that banks reduce the interest rates on loans for consumers purchasing GH. Shanghai and Fujian have reduced the cost of buying GH by rebating a certain percentage of the deed tax paid by consumers. As can be seen, the number, strength and scope of subsidies for consumers are weaker than those for enterprises at present. 

Based on the above delineation of GH incentive policies, this paper’s research on the impact of GH government policies can be translated into the following specific questions.

(i) How do subsidy policies for enterprises affect the diffusion of GH, and is it true that the larger the subsidy, the more effective it is in promoting the diffusion of GH?
(ii) How do preferential policies affect the diffusion of GH, and which preferential policies should the government prioritize to promote the proliferation of GH?
(iii) How do restrictive policies for OH affect the diffusion of GH, and is it necessary to impose more punitive policies for GH diffusion?
(iv) How do subsidy policies for consumer subsidies affect the diffusion of GH? At the same cost, which policy is more effective, consumer subsidy or enterprises subsidy? In order to study the above issues, this paper makes the following assumptions regarding government policy and the price, cost and profits associated with GH and OH.

(1) Assumptions related to GH

Assuming that there are only two categories of residential products, GH and OH, in the real estate market environment of provincial areas, the environmental friendliness of GH and OH are respectively $D_{gr}$ and $D_{tr}$. Based on the definition of GH, it is known that $D_{gr}>D_{tr}>0$. The unit construction costs of GH and OH are respectively $C_{gr}\;$ and $\;C_{tr}$, and their prices are respectively $P_{gr}$ and $P_{tr}$. According to previous studies, it can be known that $C_{gr}>C_{tr}>0$, and $P_{gr}>P_{tr}>0$.

(2) Assumptions related to government policies

A local government may adopt one or several incentive policies to promote the development of the GH industry within its jurisdiction. Firstly, assume that the increased profits for the enterprises developing GH arising from direct or indirect subsidy policies by the local government are ωS, where *ω* indicates the strength of subsidy policy used by the government. Secondly, assume that the increased benefits due to the preferred policy for the enterprises developing GH are γF, where γ is an adjustment factor to identify the extent to which policy affects the enterprises’ profits. Meanwhile, assume that the increased cost for enterprises developing OH due to the restrictive policies by local government are ρM, where ρ is the adjustment factor that indicates the strength of the policy. Lastly, assume that μI represents the total amount of subsidies that consumers can receive for purchasing GH, where μ is the adjustment factor that indicates the strength of the policies.

(3) Assumptions related to the enterprises and consumers

Assume that in a provincial market, the real estate enterprises have only two strategies, ‘developing GH’ or ‘developing OH’, with which they will earn different profits. The profits are represented as $\pi_{gr}$ for GH, and $\pi_{tr}$ for OH. Meanwhile, assuming that the total numbers of real estate enterprises and consumers are fixed, and that the consumers are heterogeneous, thus, different consumers have different degrees of green preference, which is an important indicator of consumer purchase intention, represented by θ, and that obeys a uniform distribution between 0 and 1. The payment coefficient of consumers’ green preference that indicates the fee that consumers are willing to pay for each increase in the green effort level is K [39], the net benefits for consumers to purchase GH, OH, and nothing are $U_{gr}$, $U_{tr}$, and $U_{n}$, respectively. Table 1 lists the definitions of the variables.

### 3.2. The Game Models between Real Estate Enterprises

Based on the consumer utility function proposed by Mussa (1978) [54], the consumers’ willingness to purchase green product is $\theta_{gr}D_{gr}$ [40,47]; with the introduction of the payment coefficient of consumers’ green preference K [43], it can be determined that the consumers’ willingness to purchase GH and OH are respectively $\theta_{gr}D_{gr}K$ and $\theta_{tr}D_{tr}K$. According to the calculation methods of previous scholars for consumer utility, considering government subsidies, three kinds of utility can be described as follows:

(1) $$U_{gr}=\theta_{gr}D_{gr}K-P_{gr}+\mu I;\;$$

(2) $$U_{tr}=\theta_{tr}D_{tr}K-P_{tr};\;$$

(3) $$U_{n}=0;\;$$

The above assumptions about consumer utility are based on the fact that consumers have limited rationality, which means that consumption decisions are made with the objective of maximizing utility and that consumers will only make purchases if the utility resulting from the purchase is non-negative. Based on Zhu (2011) [55] and Liu (2017) [40], the boundary value of $\;\theta_{gr}$, which represents that there is no difference between consumers’ perceived utility of GH and OH, can be determined from the association of Equations (1) and (2).

(4) $$\theta_{gr}=\frac{P_{gr}-P_{tr}-\mu I}{\left(D_{gr}-D_{tr}\right)K}$$

From the association of Equations (2) and (3), it is possible to determine the boundary value $\mathrm{of}\;\theta_{tr}$ between consumers buying ordinary houses and nothing,

(5) $$\theta_{tr}=\frac{P_{tr}}{D_{tr}K}$$

The relationship between $\theta_{gr}$ and $\theta_{tr}$ is: $0<\theta_{tr}<\theta_{gr}<1$; it reveals the degree of consumer preference for GH and OH. When $0<\theta <\theta_{tr}$, consumers will not buy any kind of residential product, when $\theta_{tr}<\theta <\theta_{gr}$, consumers will buy an ordinary residential product, and when $\theta_{gr}<\theta <1$, consumers will buy a green residential product; let the total number of consumers be 1.

The consumer demand for green housing can be determined as follows.

(6) $$Q_{gr}=\int_{\theta_{gr}}^{1}f\left(\theta \right)d\theta =1-\frac{P_{gr}-P_{tr}-\mu I}{\left(D_{gr}-D_{tr}\right)K}$$

The consumer demand for OH:

(7) $$Q_{tr}=\int_{\theta_{tr}}^{\theta_{gr}}f\left(\theta \right)d\theta =\frac{P_{gr}-P_{tr}-\mu I}{\left(D_{gr}-D_{tr}\right)k}-\frac{P_{tr}}{D_{tr}K}$$

The sum of direct benefits for the real estate enterprises in the market for developing green housing is $\pi_{gr}$:

(8) $$\pi_{gr}=(P_{gr}-C_{gr}+\omega S)\left[1-\frac{P_{gr}-P_{tr}-\mu I}{\left(D_{gr}-D_{tr}\right)K}\right]$$

Additionally, the sum of total benefits for the real estate enterprises developing OH is $\pi_{tr}$:

(9) $$\pi_{tr}=(P_{tr}-C_{tr}-\rho M)\left[\frac{P_{gr}-P_{tr}-\mu I}{\left(D_{gr}-D_{tr}\right)k}-\frac{P_{tr}}{D_{tr}K}\right]$$

Due to $\frac{\partial^{2}\pi_{gr}}{\partial^{2}P_{gr}}=-\frac{2}{\left(D_{gr}-D_{tr}\right)K}<0$, $\frac{\partial^{2}\pi_{tr}}{\partial^{2}P_{tr}}=-\frac{2D_{gr}}{D_{tr}\left(D_{gr}-D_{tr}\right)K}<0$, the profits for the real estate enterprises from developing GH and OH are convex functions with respect to $P_{gr}$ and $P_{tr}$, respectively. Thus, the profit maximization conditions are: $\frac{\partial \pi_{gr}}{\partial P_{gr}}=0$, and $\frac{\partial \pi_{tr}}{\partial P_{tr}}=0$. By associating them, the optimal prices of green housing and OH are found as:

(10) $$P_{gr}=\frac{2D_{gr}K\left(D_{gr}-D_{tr}\right)+\left(2D_{gr}-D_{tr}\right)\mu I+D_{gr}\left(2C_{gr}+C_{tr}+\rho M-2\omega S\right)}{4D_{gr}-D_{tr}}$$

(11) $$P_{tr}=\frac{D_{tr}K\left(D_{gr}-D_{tr}\right)+2D_{gr}C_{tr}+D_{tr}C_{gr}-\mu ID_{tr}-D_{tr}\omega S+2\rho MD_{gr}}{4D_{gr}-D_{tr}}$$

From Equations (10) and (11), it can be seen that the optimal price set by real estate enterprises is influenced by the cost of real estate development, the greenness of the housing, the marginal ability of consumers to pay, and the government’s incentive policies. In the market environment, real estate enterprises will adjust the sales price to the above price in order to maximize profits. Equations (10) and (11) are brought into Equations (6) and (7). It can be determined that under the optimal price strategy, the consumer demands for GH and OH are:

(12) $$Q_{gr}=\frac{2D_{gr}K\left(D_{gr}-D_{tr}\right)+\left(D_{tr}-2D_{gr}\right)C_{gr}+D_{gr}C_{tr}+\left(2D_{gr}-D_{tr}\right)\mu I+\left(2D_{gr}-D_{tr}\right)\omega S+\rho MD_{gr}}{\left(4D_{gr}-D_{tr}\right)\left(D_{gr}-D_{tr}\right)k}$$

(13) $$Q_{tr}=\frac{D_{gr}\left[D_{tr}K\left(D_{gr}-D_{tr}\right)+D_{tr}\left(C_{gr}-\omega S\right)-\left(2D_{gr}-D_{tr}\right)\left(C_{tr}+\rho M\right)-\mu ID_{tr}\right]}{\left(4D_{gr}-D_{tr}\right)\left(D_{gr}-D_{tr}\right)D_{tr}k}$$

The direct economic benefits for real estate enterprises to develop GH and OH are respectively:

(14) $$\pi_{gr}=\frac{{\left[2D_{gr}K\left(D_{gr}-D_{tr}\right)-\left(2D_{gr}-D_{tr}\right)\left(C_{gr}-\omega S-\mu I\right)+D_{gr}(C_{tr}+\rho M)\right]}^{2}}{{\left(4D_{gr}-D_{tr}\right)}^{2}\left(D_{gr}-D_{tr}\right)k}$$

(15) $$\pi_{tr}=\frac{D_{gr}{\left[D_{tr}K\left(D_{gr}-D_{tr}\right)+D_{tr}\left(C_{gr}-\omega S-\mu I\right)-\left(2D_{gr}-D_{tr}\right)\left(C_{tr}+\rho M\right)\right]}^{2}}{{\left(4D_{gr}-D_{tr}\right)}^{2}\left(D_{gr}-D_{tr}\right)D_{tr}k}$$

**Figure 2:**
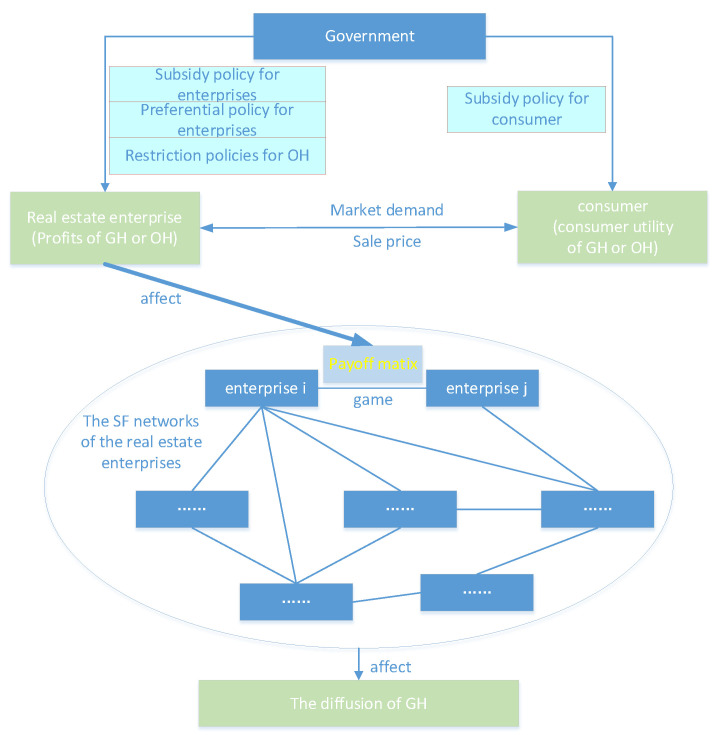
Diagram of the networked evolutionary game for GH diffusion.

In the above analysis, $\pi_{gr}$ and $\pi_{tr}$ represent the sum of the profits for the enterprises developing GH and OH, respectively, in a certain market range. For individual enterprises, their profits are also related to the number of enterprises choosing the corresponding strategies. Assuming that the number of enterprises choosing the GH strategy is $N_{gr}$ and the number of firms choosing the OH strategy is $N_{tr}$, the revenue from developing GH for enterprise *i* is $\frac{\pi_{gr}}{N_{gr}}$ and the revenue from developing OH for enterprise *j* is $\frac{\pi_{tr}}{N_{tr}}$. The payment matrix of the game between enterprise *i* and enterprise *j* is shown in the Table 2. The diagram of the networked evolutionary game for GH diffusion is shown in Figure 2.

### 3.3. Networked Evolutionary Dynamics Model

The game relationship among players in the evolutionary game model mentioned above reflects the information interaction or market competition existing among the real estate enterprises, which in reality can be more specifically expressed as a network structure. Considering that some large real estate enterprises have a large number of partners or competitors, and some small enterprises have few in the real estate market, we constructed a scale-free (SF) network to study the diffusion of GH. The SF network constructed by the real estate enterprises is based on the BA SF network [56]; the degree of distribution of scale-free networks follows a power law, meaning that a few hub nodes have an extremely large number of connections, while most nodes have only a small number of connections. Let the network of real estate enterprises be *G(V,E)*, where *V* is the set of network nodes, each node is a specific real estate enterprise, *E* is the set of network-connected edges, each edge represents the game relationship between two node enterprises, and the number of nodes is expressed as *N*. Considering that the communication between enterprises is mutual behavior, there is no direction, so the network *G(V,E)* is an undirected network.

In the scale-free network model, the game object of a node enterprise is the neighboring nodes with which it has connected edges, and in each game, all nodes play this game with their neighbors and accumulate the gains. In the strategy evolution, the stochastic strategy evolution rule is considered based on the Fermi rule, i.e., individual *i* will randomly choose a neighbor *j* for strategy comparison, and if the neighbor’s current round gain is higher than its own gain, in the next round it will imitate the neighbor’s current round strategy with a certain probability. This imitation probability is calculated according to the Fermi function in statistical physics.

(16) $$W_{s_{i}\leftarrow s_{j}}=\frac{1}{1+exp\left[\frac{\left(U_{i}-U_{j}\right)}{k}\right]}$$

where: $s_{i}$ denotes the strategy taken by the individual enterprise *i* and, $U_{i}$ is the gain by enterprise *i* in this round. $s_{j}$ is the strategy of individual enterprise *j*, and $U_{i}$ is the gain by enterprise *j* in this round. The function indicates that when the gain of enterprise *i* in this round is lower than the gain of enterprise *j*, enterprise *i* will easily accept the strategy of enterprise *j*. However, when the gain of *i* is higher than the gain of *j*, it will also learn the strategy of enterprise *j* with a weak probability. This irrational choice of the individual is portrayed by *k*; values of *k* closer to 0 mean that the individual’s irrational choice tends to zero, and the strategy update is determined. If the gain of the comparison object is higher than its own, it will definitely choose to learn, or vice versa, it will stick to its original strategy; a *k* value tending toward infinity means that the individual is in a noisy environment, unable to make rational decisions, and can only update its own strategy randomly. The potential adopter node *i*, after choosing a learning strategy with probability *W*, will reconnect with other nodes in the network with probability $\gamma_{ij}$. In this paper, the reconnection mechanism with preference is used to determine the outgoing connection *j* of a node, and the random probability $\gamma_{ij}$ can be expressed as:

(17) $$\gamma_{ij}=\sum_{i\in G}\frac{U_{j}^{\propto }}{U_{i}^{\propto }}$$

where, $U_{i}$ is the gain of node *i*, $U_{i}$ is the gain of node *j*, ∝=0 means this link does not have any preference tendency and is a random link; the larger ∝ is, the more obvious the preference tendency is. According to the above rules, the node enterprise determines its own revenue in this round by forming patterns with other neighboring nodes, and then learns the game strategies of other nodes by comparing the revenue, and the game and learning among nodes realize the diffusion of ‘developing GH’ strategy on the whole network model, so as to simulate the diffusion of GH in the market. This is to simulate the diffusion of GH in the market.

## 4. Simulation Analysis

### 4.1. Data and Parameters

According to our data from the National Bureau of Statistics, the number of real estate development enterprises in China was 99,544 in 2019, while the number of real estate law enterprises in each province varied greatly. Due to the long construction and sales cycles of real estate projects, the number of enterprises in the provincial market that are simultaneously developing and building real estate projects is generally between 300 and 800. To facilitate simulation tests, the number of nodes in the network of real estate enterprises was assumed to be 500.

In order to produce simulation results in conformity with the actual situation in a province with lagging GH development in China, we selected Heilongjiang, a northeastern province in China, as the reference object, where the average selling price of OH, according to statistics for 2021, was CNY 2197–9705 per square meter. Based on the current average price of real estate, we assumed that the unit cost of OH is CNY 5200/m^2^, (approx. EUR 718/m^2^) and the unit cost of GH can be assumed to be CNY 6300/m^2^, as the cost premium for green houses ranges from 5% to 21% [57].

The environmental friendliness represents the overall effectiveness of GH, which includes energy efficiency, environmental protection, pollution reduction over the building life cycle and provision of a healthy and comfortable environment to users [58]. Based on Liang (2019) [59], we can assume that the environmental friendliness of GH and OH is 0.9 and 0.76, respectively, and the marginal payment coefficient for GH is CNY 7700/m^2^ (approx. EUR 1063/m^2^).

Currently, among the provinces implementing a monetary subsidy policy, Shanghai has the highest financial subsidy, with a funding amount of CNY 10–60/m^2^; in particular, for prefabricated assembly rates of 25%, the funding amount is increased to CNY 100/m^2^; the amount is lower in the western region, with Shaanxi Province’s incentive for GH being CNY 10–20/m^2^. The extent of reduction in the urban infrastructure facility fee varies from province to province due to different levy standards. In Hainan, it can be reduced by CNY 30–88/m^2^ with a rating of two stars or above, and the reduction is CNY 15–30/m^2^ in the Inner Mongolia Autonomous Region. Considering the superimposed effect of multiple financial subsidies, the increased profits for enterprises, S, is assumed to be CNY 120/m^2^.

The preferential policy can enhance the reputation and market competitiveness of a real estate development company, translating into potential benefits [39,53]. Based on previous studies, we assumed that the increased benefits for enterprises due to the preferred policy *F* is CNY 10/ m^2^.

In the provinces that have implemented restriction policies for OH, the government often requires a certain percentage of new housing to be green, meaning it is more difficult for real estate enterprises to obtain land to develop OH, and the cost of OH will be increased. Considering the environmental pollution tax and the cost of weak product competitiveness and opportunity costs [60], based on previous studies, the additional cost, *M,* for developing OH is set at CNY 75/m^2^.

For consumers, Anhui Province explicitly requires that financial institutions lower interest rates by 0.5%. Xiamen City in Fujian Province offers a 1% to 3% reduction in the purchase tax for consumers. In conjunction with the previous analysis of consumer subsidies and to facilitate comparison with the effectiveness of the implementation of enterprise subsidy policies, the value of the sum of the various types of subsidies received by consumers, *I*, for the purchase of GH is set at CNY 120/m^2^. The initial values of all variables are reported in Table 3.

To estimate the effects of government policies on the diffusion of GH, we varied the strength of the four government policy tools in the simulation. The adjust factors ω, γ, ρ and μ are set to 0.2, 0.4, 0.6, 0.8 and 1.0, and the impact of different strengths of policy on the diffusion of GH by the change of the factors is analyzed.

The results are the average of 50 independent simulation experiments considering the random factors in the process of evolution games.

### 4.2. Simulation Analysis of the Influence of Government Policy on GH Diffusion

#### 4.2.1. The Influence of Enterprise Subsidy Policies S on GH Diffusion

**Figure 3:**
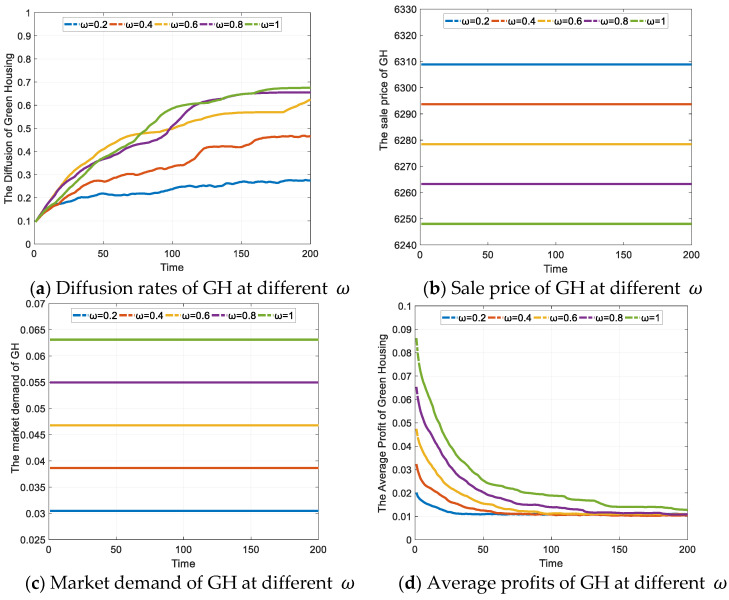
The effect of enterprises subsidy on GH diffusion (Unit: CNY).

The simulation results of the effect of different levels of enterprise subsidy policies on the diffusion rate of GH are shown in Figure 3. Figure 3a shows the increase in the diffusion rate of GH under different subsidy levels; it can be seen that the subsidy policies can effectively increase the diffusion of GH development strategies within a group of enterprises, and that the efficient transmission of information on the scale-free network and the learning ability of the nodes accelerate the diffusion of GH strategies. As *ω* increases, the diffusion rate of GH increases, as shown by the different colored curves in the figure, which can eventually reach 0.6752 when ω is 1. However, the diffusion curves in Figure 3a show that although the increase in government subsidies is fixed, the diffusion of GH shows a different increase; the magnitude of increase in diffusion rates gradually becomes narrow as the subsidy increases. With both prices and demand increasing, the diffusion rate of GH does not increase significantly at ω taken as 0.6, 0.8 and 1. The maximum diffusion rates of GH at different ω are shown in Table 4. The implication is that a moderate subsidy policy can effectively increase the willingness of enterprises to develop GH, but the marginal effect of the incentive policy diminishes as the subsidy increases.

This phenomenon can be explained as follows: firstly, the subsidy policies for enterprises have an impact on the market price and demand for green homes. As shown in Figure 3b, when market demand for green homes intensifies as subsidies grow, the enterprises will increase the sales price of GH in order to maximize revenue, as shown in Figure 3c. The total profits from GH, which are determined by the average profits of single enterprises and the number of GH enterprises, will increase due to growth in demand and the price of GH. As shown in Figure 3d, for *ω* = 0.2, 0.4, 0.6, and 0.8, there is no significant increase in the average profit from GH, because the number of GH enterprises has increased rapidly, and the increased profits due to the subsidy policy are captured by the new entrants. Additionally, when *ω* = 1, the average profit from GH rises more significantly, meaning that there is no significant increase in the number of GH enterprises. It can be seen that the incentive effect of the subsidy policy is influenced by industry competition and the scale-free network structure, which in turn affects the average profit from GH and facilitates the proliferation of GH as shown by the curve in Figure 3a.

In the real estate marketplace, enterprises that enter the GH market early with the support of government subsidy policies will earn higher profits and gain a competitive advantage. The success of some larger or stronger enterprises (which can be regarded as nodes with more connected edges in a scale-free network) will encourage more enterprises to choose GH. However, the enterprises entering the marker later will face stronger competition and earn a lower level of profits. When the profit is less than that of OH due to the competition, some of the enterprises will abandon GH strategy, even if they are eligible for the government subsidies.

#### 4.2.2. The Effect of Preferential Policy γ on GH Diffusion

**Figure 4:**
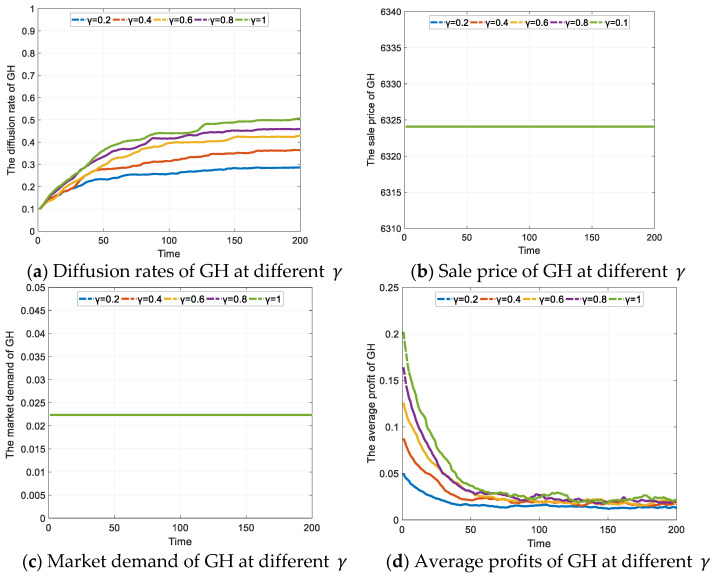
The effect of preferential policy on GH diffusion (Unit: CNY).

As shown in Figure 4a, the government’s preferential policy can also effectively increase the diffusion rate of GH, which can reach a maximum of about 0.506 under the assumptions in the paper. The maximum diffusion rates of GH at different γ are shown in Table 5. Although the diffusion rate of GH is effectively increased, the incentive effect is less desirable than that of the subsidy policy due to the smaller benefit afforded by the preferential policy. Meanwhile, the curves in the figure indicate that the increase in GH diffusion, at γ  = 0.8 and γ = 1, is lower than at γ = 0.2, γ = 0.4 and γ = 0.6. This means that the government may also face a problem with marginal effects, which are decreasing under the preferential policy; as the policy grows stronger, its incentive effect will diminish.

For consumers, the preferential policy does not have a direct impact on the benefits of purchasing GH, so the market demand for GH does not change under different strengths of the policy, and the stability of demand prevents the enterprises from adjusting the sales price of GH, as shown in Figure 4b,c. However, the preferential policy can bring a competitive advantage to the GH project and enhance the profitability of the enterprises. For example, prioritizing administrative approvals can reduce the administrative costs and shorten the development cycle of GH projects. The advantages created by the implementation of similar measures can be translated into financial returns for the GH enterprises. As shown in Figure 4d, in the early stage of the games, the GH enterprises will obtain higher profits due to the preferential policy. Subsequently, as γ become larger, the number of GH enterprises increases rapidly, and the average profit begins to decline. In the later stages of the games, the curves show fluctuations, which are due to industry competition, which can accelerate the weakening or disappearance of the advantage, leading to the rapid withdrawal of some enterprises from the GH market.

In general, the preferential policy for GH can still increase the average profit earned by GH enterprises, and accelerate the diffusion of GH, but its incentive effect is relatively weak compared to the direct financial subsidy policy. This corresponds to the actual situation in the northeastern and northwestern provinces of China; although these provinces have introduced various preferential policies to encourage real estate enterprises to build GH, the development of GH is still relatively slow.

#### 4.2.3. The Effect of the Restriction Policy M for OH on GH Diffusion

**Figure 5:**
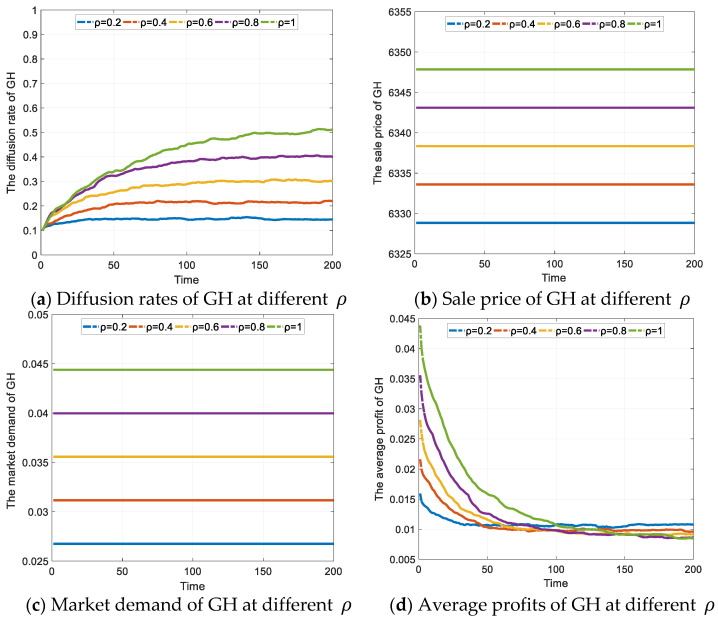
The effect of OH restriction policy on GH diffusion (Unit: CNY).

As shown in Figure 5a, the shapes of the curves reflect the growth of GH diffusion rate as the policy strength increases, with the maximum diffusion rate of 0.5137 at ρ = 1. The maximum diffusion rates of GH at different ρ are shown in Table 6. Land restrictions or taxes have led to higher costs for OH, encouraging more enterprises to choose GH. In the games, the structural features of the scale-free network allow information to spread more efficiently, and the diffusion of GH is faster as the value of *ρ* increases. Meanwhile, the increase in the GH diffusion rate at ρ = 0.8 and ρ = 1 is higher than that at ρ = 0.2, ρ = 0.4 and ρ = 0.6, which indicates that real estate enterprises are more sensitive to high-intensity restrictive policies, and the stronger the restrictions on OH, the stronger the willingness of enterprises to develop GH.

According to the rise in the diffusion of GH, the restriction policy for OH is less effective than the subsidy policy for GH, mainly because the existing restriction policy has less binding power and does not increase the cost of developing OH for enterprises. However, the policy can still increase the demand for GH, as shown in Figure 5b, and with a rise in demand, the enterprises increase the selling price of GH, as shown in Figure 5c. Hence, the changes in price, demand, and the number of GH enterprises will directly affect the average return on GH, as shown in Figure 5d. In the early games, the increase in the value of ρ will cause growth in the average profit from GH. Later, as the GH strategy spreads, the number of GH enterprises increases rapidly, leading to a rapid decline in the average profit from GH, and the larger the value of ρ, the more pronounced this decline is. In the later stages of the game, the average returns from GH are relatively stable, at which point, although the returns are lower, the enterprises will still choose to develop GH as long as the profits is higher than OH.

In the actual market environment, the implementation of a restrictive policy will make the cost of OH increase significantly and inevitably lead to an increase in the price of OH, resulting in an increase in demand for GH. That means whether consumers buy GH or OH, they will pay a higher price. The more restrictive the policy, the higher the cost of home ownership for consumers. Therefore, in a healthy real estate market, the restrictive policy should be used with caution.

#### 4.2.4. The Effect of the Subsidy Policy I for Consumers on GH Diffusion

**Figure 6:**
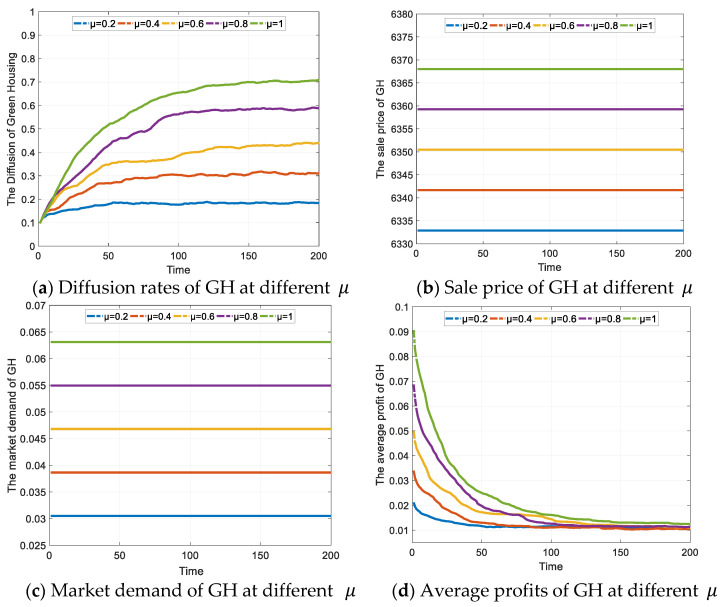
The effect of consumers subsidy policy on GH diffusion (Unit: CNY).

Figure 6a shows the diffusion of GH under different policy strengths in the games. The maximum diffusion rates of GH at different μ are shown in Table 7. The simulation results show that the increase in the diffusion rate of GH is small when the consumer subsidy is low. As the subsidy increases, the structure of the scale-free network accelerates the diffusion of information, accelerating the diffusion of GH. At the same time, as the probability of success of policy learning is determined according to the Fermi function (function 16), the greater the difference between the returns of the two policies, the higher the probability of success of policy learning, resulting in a further increase in the diffusion rate of GH, reaching 0.7097 at μ = 1, which is slightly higher compared to subsidy policy for enterprises with the same amount of subsidy. However, the diffusion rates of GH are all lower than that from the enterprises subsidy policy. In this regard, we can argue that, on condition that the subsidies are below CNY 120/m^2^, the enterprise subsidies are more effective than consumer subsidies in promoting the diffusion of GH among the enterprises.

In general, the consumer subsidy policy will increase the demand for GH, as is shown in Figure 6b. In order to maximize profits, the real estate enterprises will increase the sales price of GH, as shown in Figure 6c. In the early games, the enterprises with ‘developing GH’ strategy will gain higher profits, and the others with ‘developing OH’ strategy will learn the strategy from the game player, thus facilitating the diffusion of GH. In this process, some enterprises are more influential than others, and when these enterprises earn higher profits from developing GH, the advantages of this strategy will spread faster due to the structural characteristics of the scale-free network, encouraging a larger number of enterprises to enter the GH market, with the result that competition between them causes the average profit from GH to decline subsequently, as shown in Figure 6d. When the profitability of developing GH is lower than that of OH, some enterprises will choose to develop OH again. Thus, the strength of consumer subsidies and the intensity of competition in the industry lead to a steady state in the number of GH firms, which is reflected in the relatively stable diffusion rate of GH in the last games in Figure 6a.

In the real estate market, the increase in subsidies to consumers will cause a rise in the price of both ordinary and green homes, although the demand for ordinary homes will decrease as a result. If the price of GH rises too high, consumers will find it difficult to afford them and switch to OH, thus forming renewed demand for OH and creating an obstacle to the diffusion of GH.

## 5. Conclusions

This paper classifies the incentive policies into four categories, such as subsidy policies for enterprises to develop GH, restrictive policies for enterprises that develop OH, priority policies for GH and subsidy policies for consumers. Using an evolutionary game model on a complex network, it analyzes the impact of various government policies on market demand for GH and the profits of enterprises, reveals the mechanisms of the various policies, and analyzes the incentive effects through simulation. The study shows that:

(1) The subsidy policy for enterprises can effectively promote the diffusion of GH, but as the amounts of financial subsidies increases, the incentive effect of the policy gradually decreases, which can eventually reach 0.6752. The simulation results show that, within a certain range, an increase in the amounts of subsidies targeted at enterprises can effectively increase the demand for GH and the profits of the enterprises, which are conducive to the expansion of GH scale. This is in line with He (2021) [39] and Feng (2020) [42]. However, the effect of direct or indirect subsidies does not increase equally with the increase in the subsidy level. After the subsidy rises to a certain level, the real estate enterprises will be less sensitive to the subsidy policy; ‘market interest’ and ‘competition pressure’ are among the key factors that influence the adoption of ‘green strategies’ [60].
(2) The preferential policy can be effective in promoting the development of GH, the diffusion rate can reach a maximum of 0.506, and as the diffusion rate rises higher, the incentive effect will be weaker. The preferential policies do not directly affect the pricing and market demand for GH, but they can provide additional potential benefits for the GH enterprises, which will gain a competitive advantage over the ordinary. However, the advantage will diminish or disappear with the increase in the numbers of GH enterprises, because the enterprises entering the GH market later can hardly gain any priority from the policy.
(3) The restrictive policies for OH can be an effective driver to push up the diffusion rate of GH; the maximum diffusion rate is 0.5137. Simulation results show that the implementation of restrictions or penalties on OH increases demand for GH and raises its price, resulting in a rapid increase in the number of GH enterprises, and the stronger the restrictions on OH, the higher the diffusion rate of GH.
(4) The subsidy policy for consumers can be effective in promoting the diffusion of GH, but if the subsidy is below a certain level, its effect is less powerful than that of the enterprises subsidy policy. Under the assumptions of this paper, at a lower subsidy level, i.e., less than CNY 120/m^2^, the incentive effect of the consumer subsidy policy is weaker than that of the enterprise subsidy policy, and if the subsidy is above 120 CNY/m^2^, the former is slightly more effective than the latter. These conclusions are somewhat different from the research results of Olubunmi (2016) [19] and He (2021) [39], which suggested that the demand-side incentives are more effective in promoting commercial green buildings.

The expansion in GH scale and the improvement of energy saving effects are very important for energy savings and emission reductions in construction industry and the improvement of people’s living environment. Many provincial governments in China have emphasized the development of GH in the 14th Five-Year Plan. However, the current Chinese real estate market has become more complicated due to the impact of government regulatory policies and financial supply, which require that local governments formulate and implement more scientific, detailed, and targeted policies and measures to effectively promote the diffusion of GH. During the process of policy design and implementation, the following aspects should be considered.

Firstly, to avoid over-reliance on subsidies, the subsidies for GH enterprises should have an appropriate standard based on the development level of GH in the area. At present, the enterprise subsidy is a common incentive policy for GH in China; only certain provinces in the northeast and northwest do not yet have a relevant policy. For the provinces with a low level of GH development, the subsidy policies will be a good option worth paying for, especially for the purpose of boosting the rate of diffusion of GH in a short time. Meanwhile, to reduce the financial pressure, indirect subsidies, such as floor area ratio incentives and loan interest rate concessions, may be a better choice for these local governments. For the provinces with a high level of GH development, the subsidy for enterprises to develop GH should have dynamic and flexible attributes, meaning that the government should be able to control the dependence of enterprises on subsidies by adjusting the strength of the policy. In addition, the competition among enterprises and the influence of large real estate enterprises should be considered in the formulation and implementation of policy to improve the effectiveness of incentives to develop GH.

Secondly, the preferential policies can be widely used and will be more effective when combined with other policies. Unlike the subsidy policy, the priority policy can be applied broadly, because it is not limited by government budgets. Considering that the assumptions in the previous section are set in the context of the provinces with lower housing prices, in areas with higher housing prices, larger subsidies will be needed to achieve good results. The advantages of the policy are obvious, and it can be an effective policy tool to improve the diffusion of GH. However, it should be noted that the key factor affecting the effectiveness of the policy is how much of a competitive advantage it can provide to the GH enterprises; the incentive effect of the policy will be weakened by the increase in the diffusion rate of GH. Therefore, after GH development has risen to a certain level, the incentive effect can be further enhanced by using it in conjunction with other policies, such as consumption guidance and floor area ratio incentives.

And again, the restriction policy for OH can be a powerful tool to promote the proliferation of GH, but it needs to be used with caution. In the case that consumers still have a certain demand for OH, excessive restrictions or penalties will not only cause the price of OH rise, but also cause the price of GH increase significantly, which is obviously not in accord with the current control concept of the Chinese central government for the real estate market. Therefore, the local governments should apply principles of scrupulousness in the implementation of the policy to avoid the situation of high prices with shrinking demand. In view of the current market environment, to maintain the stable and healthy development of the real estate industry, it is not advisable to introduce punitive regulatory measures or stronger restrictive policies on OH.

Finally, the consumer subsidy should be offered by local governments to strengthen the demand for GH and thereby foster a favorable environment for the industry’s development. At present, only a few provinces in China have implemented the subsidy policy for consumers, whereas most provinces choose to subsidize the enterprises that can effectively increase the diffusion rate of GH in the early development stage. Additionally, as the subsidy rises to a certain level, the consumer subsidy policy will be more effective in terms of forming long-lasting and stable market demand for GH. However, with the development of green buildings, incentive policies will be revised and adjusted continuously, and the market may play an increasingly important role [61]. To this end, the government should focus on cultivating a mature market, raising public awareness of GH [62], effectively expanding market demand for GH, and ultimately relying on the market supply and demand mechanism to achieve the overall greening of residential products. For this reason, the government should focus on raising public awareness, expanding market demand for GH, and cultivating a mature market [63]; ultimately, the green transition for the real estate industry will be realized by the means of optimizing and improving the supply and demand mechanism of the GH market.

‘Turn to green’ is not only an effective solution for building energy efficiency, but also a way to a low-carbon, environmentally friendly, and healthy life. For China, as an effective external incentive, government policies are necessary in the early stages of GH development. However, as the scale of GH expands, local governments need to adopt appropriate incentives based on the level of economic development, population size, consumer preferences, the geographic environment, and real estate market development trends, and adjust the policy intensity in a timely manner to cultivate a favorable environment for the development of the GH industry. Therefore, when implementing incentive policies for GH, local governments should set reasonable subsidy levels, taking into account the regional economic environment, pay attention to incentives on the demand side of GH, and further strengthen priority policies for green homes in terms of land supply and administrative approval, while cautiously using restrictive and punitive policies for traditional homes, so as to promote the healthy growth of the green home market.

The evolutionary game model on the complex network has provided a better explanation of how government policy affects the diffusion of green homes with its influence on the price of and demand for GH and the profits of enterprise; meanwhile, it also reflects the effect of information exchange and competitive relationships between firms on the effect of policy incentives. This shows the applicability and superiority of this model in studying the diffusion of green products or technologies. However, the assumption of consumer utility in the model is not perfect, in that the price of and market demand for green housing do not change with the game between firms, but only increase or decrease with the change of policy strength, which is different from the actual market environment. In subsequent research, we will construct a two-tier network evolutionary game model based on consumers and enterprises to discuss this issue in more depth. In addition, due to the limitation of space, this paper did not study the effects of network size and network type on policy effects: for example, whether the incentive effect of government policies enhances or weakens with the increase in the number of network nodes, and whether there are large differences in the incentive effect of policies in different network environments such as scale-free networks, ER random networks, and small-world networks. In addition, the paper did not discuss the effects of factors such as consumer preferences and the green degree of residential products on the incentive effects of government policies, and the incentive effects of multiple policies implemented simultaneously were not included in the scope of the simulation analysis. In the subsequent study, we will focus on analyzing the effects of changes in network topology on policy incentive effects and the diffusion of GH and further discuss the effects of a mixture of multiple policies on the diffusion of GH, thereby suggesting optimization of the policy system for local governments to promote GH development.

## Figures and Tables

**Table 1:** Definition of variables.

Variables	Definition
$D_{gr}$	The environmental friendliness of GH
$D_{tr}$	The environmental friendliness of OH
$C_{gr}$	The unit construction costs of GH
$C_{tr}$	The unit construction costs of OH
$P_{gr}$	The unit price of GH
$P_{tr}$	The unit price of OH
S	Subsidy for the enterprises by local government
F	Increased benefits due to preferential policy for enterprises
M	Increased cost for OH due to restrictive policies
I	Subsidy for consumers by local government
$\pi_{gr}$	The profits of enterprises by developing GH
$\pi_{tr}$	The profits of enterprises by developing OH
θ	Degrees of green preference of the consumers
K	The payment coefficient of consumers’ green preference

**Table 2:** The payoff matrix for both enterprises.

Enterprise and Their Strategies		Enterprise *j*	
		Developing GH	Developing OH
Enterprise *i*	Developing GH	$\frac{\pi_{gr}}{N_{gr}}$ $,\;\frac{\pi_{gr}}{N_{gr}}$	$\frac{\pi_{gr}+\gamma F}{N_{gr}}$ $,\;\frac{\pi_{tr}}{N_{tr}}$
	Developing OH	$\frac{\pi_{tr}}{N_{tr}}$ $,\;\frac{\pi_{gr}+\gamma F}{N_{gr}}$	$\frac{\pi_{tr}}{N_{tr}}$ $,\;\frac{\pi_{tr}}{N_{tr}}$

**Table 3:** The initial values of all variables.

*N*	*D_gr_*	*D_tr_*	*C_gr_*	*C_tr_*	*K*	*G*	*F*	*M*	*I*
500	0.9	0.76	6300	5200	7700	120	10	75	120

Unit: CNY.

**Table 4:** The diffusion rate of GH at different ω.

ω	0.2	0.4	0.6	0.8	1
The diffusion rate of GH (max)	0.2767	0.4684	0.6243	0.6555	0.6752

**Table 5:** The diffusion rate of GH at different γ.

γ	0.2	0.4	0.6	0.8	1
The diffusion rate of GH (max)	0.2865	0.3648	0.429	0.4596	0.506

**Table 6:** The diffusion rate of GH with different ρ.

ρ	0.2	0.4	0.6	0.8	1
The diffusion rate of GH (max)	0.1543	0.221	0.3082	0.4061	0.5137

**Table 7:** The diffusion rate of GH with different μ.

μ	0.2	0.4	0.6	0.8	1
The diffusion rate of GH (max)	0.1889	0.3186	0.4405	0.5910	0.7097

## Data Availability

Data is contained within the article.
